# Morphological and genetic heterogeneity of synchronous multifocal lung adenocarcinoma in a Chinese cohort

**DOI:** 10.1186/s12885-021-07892-8

**Published:** 2021-02-18

**Authors:** Donglin Zhu, Dan Cao, Minghong Shen, Jinghuan Lv

**Affiliations:** 1 Department of Thoracic Surgery, The Affiliated Suzhou Hospital of Nanjing Medical University, Suzhou Municipal Hospital, Gusu School, Nanjing Medical University, Suzhou, 215002 China; 2 Department of Pathology, The Affiliated Suzhou Hospital of Nanjing Medical University, Suzhou Municipal Hospital, Gusu School, Nanjing Medical University, Suzhou, 215002 China

**Keywords:** Synchronous multifocal lung cancer (SMLC), Synchronous multifocal primary lung cancer (SMPLC), Morphological assessment, Multiplex genotypic analysis

## Abstract

**Background:**

Synchronous multifocal lung cancer (SMLC) is diagnosed with increasing frequency in clinical practice globally. Due to innate variation in clinical management and outcome, it is vital to properly distinguish between synchronous multifocal primary lung cancer (SMPLC) and intrapulmonary metastasis (IM). The pathologic features and principal classification criteria of multifocal lung cancer remain unclear. Our objective was to evaluate the diagnostic value of histological morphologic features and driver gene mutations in SMLC classification.

**Methods:**

We collected a unique cohort of Chinese patients with SMLC, and fully explored the morphologic, immunohistochemical, and molecular features of the disease. Twenty-one SMLC patients with a total of 50 tumours were included in our study. The pathological features that were presented by these patients were analysed, including the tumours location, tumours size, pathological types, predominant pattern of adenocarcinoma, and immunohistochemical staining. We conducted molecular testing of nine driver oncogenes that are associated with lung cancer, namely, EGER, KRAS, BRAF, NRAS, ALK, ROS1, RET, HER2, and PIK3CA.

**Results:**

According to the Martini-Melamed classification and refined standard, 8 and 17 patients, respectively, were considered to have SMPLCs. Gene mutations were identified in 18 tumours (36%). Twelve patients had different gene mutations.

**Conclusions:**

We demonstrate that conventional morphological assessment is not sufficient to clearly establish the clonal relationship of SMPLCs. Instead, the evaluation of histological subtypes, including nonmucinous adherent components, is required. Multiplex genotypic analysis may also prove to be a useful additional tool.

## Background

With the increased use of high-resolution computed tomography (CT) in lung cancer screening, there has been a substantial rise in the apparent incidence of pulmonary nodules, particularly synchronous multifocal lung cancers (SMLCs) [1, 2]. The presence of more than one pulmonary nodule raises a critical clinical question: Do such nodules arise from the same clone or do they represent multiple lung cancers with independent lineages? The accurate distinction between synchronous multifocal primary lung cancers (SMPLCs) and intrapulmonary metastasis (IM) is vital since it guides therapeutic management. However, it remains challenging [3].

The original diagnostic criteria for SMLCs were defined by Martini and Melamed in 1975 [4]. These researchers mainly considered clinical and pathological characteristics, such as histological classification, tumours location, presence/absence of adenocarcinoma in situ (AIS), and lymph node metastases. A primary limitation of their approach is that only the major histological tumour type, such as adenocarcinoma or squamous cell carcinoma, is considered. However, the morphologies of the primary and metastatic foci should be consistent, without considering the histological subtypes or molecular features of the tumour.

Revisions to the histologic classification that was published by the World Health Organization (WHO) in 2015 described five main morphologically distinct subtypes of invasive adenocarcinoma: lepidic, acinar, papillary, micropapillary, and solid [5]. The solid and micropapillary subtypes usually have a poor prognosis, while the lepidic subtype usually has a more favourable outcome [6, 7].

Molecular typing has also become much more prevalent in pathological diagnosis. Various oncogene mutations are implicated in lung cancer. These mutations often play a decisive role in targeted therapy and are likewise important in determining tumour origin.

It is widely believed that while comprehensive histological assessment (CHA) has disadvantages, it can nonetheless largely address the issue of cancer classification satisfactorily. However, there is still no consensus on the proper classification of SMLC. The overall landscape of SMLC lesions remains poorly defined.

A cohort of 21 patients with SMLCs were reviewed retrospectively address these various problems; to explore the correlation among the histological morphology, subtypes, and driver gene mutation status of SMLC lesions; to identify their potential internal association; and to provide reliable clues regarding the classification of SMLCs. The histological subtypes, immunohistochemical phenotypes, and molecular characteristics were determined. The exploration of pathological and genetic features in each tumours lesion seemingly provides important additional information that is relevant to the accurate distinction between SMPLC and IM.

## Methods

### Patients

The patients who were included in this study underwent pulmonary resection at the Affiliated Suzhou Hospital of Nanjing Medical University between January 2018 and December 2019. A total of 50 distinct lung tumours that were removed from 21 patients with at least two lesions were selected for histopathologic examination. No patient had received treatment before surgery. Clinical demographic characteristics (age, sex, smoking history, tumour size, pathologic classification, histological subtypes, clinical stages, and lymphatic metastases) were obtained from electronic medical records (Table 1).

**Table 1:** Clinicopathologic characteristics of patients with SMLC

Case	Sex	Age	Tumor	Site	Size (cm)	LM	Type	Subtype(%)					Histologic type
								L	A	P	M	S	
1	F	62	01	LUL	2.5	N	AP	0	100	0	0	0	
			02	RUL	3.5		ASC	N/A	N/A	N/A	N/A	N/A	
2	F	51	01	RLL	0.6	N	AIS	100	0	0	0	0	
			02	RLL	0.4		AIS	100	0	0	0	0	
3	F	55	01	RUL	0.4	N	AIS	100	0	0	0	0	
			02	RML	0.8		LP	80	20	0	0	0	
4	F	43	01	RLL	0.3	N	AIS	100	0	0	0	0	
			02	RML	0.7		LP	60	40	0	0	0	
			03	LUL	0.5		AIS	100	0	0	0	0	
			04	LLL	0.4		AIS	100	0	0	0	0	
5	F	51	01	RML	0.6	N	AIS	100	0	0	0	0	
			02	RLL	0.5		LP	70	30	0	0	0	
			03	RLL	0.4		AIS	100	0	0	0	0	
6	F	75	01	RLL	1.5	N	AP	20	80	0	0	0	
			02	RUL	1.7		AP	0	70	20	10	0	
7	F	47	01	RLL	1.3	N	AP	0	100	0	0	0	
			02	RUL	0.5		AIS	100	0	0	0	0	
8	M	74	01	RUL	4	N	AP	0	50	0	0	50	
			02	RLL	0.7		MA	N/A	N/A	N/A	N/A	N/A	
9	F	32	01	RLL	0.8	N	AP	0	100	0	0	0	
			02	RLL	0.5		AIS	100	0	0	0	0	
10	M	78	01	RUL	2.0	N	SP	0	0	0	0	100	
			02	RUL	1.4		AIS	100	0	0	0	0	
11	M	67	01	RLL	0.9	N	AIS	100	0	0	0	0	
			02	RLL	0.8		LP	60	20	20	0	0	
12	F	61	01	RUL	1.2	N	AP	40	60	0	0	0	
			02	RUL	0.9		LP	80	20	0	0	0	
			03	RUL	0.9		AIS	100	0	0	0	0	
13	M	65	01	LUL	1.2	N	LP	40	40	10	10	0	
			02	LUL	1.1		LP	80	15	5	0	0	
			03	RUL	0.5		AIS	100	0	0	0	0	
			04	RLL	2.0		PP	20	30	40	10	0	
14	M	87	01	LLL	1	N	AP	20	50	0	0	30	
			02	LLL	1.3		AP	0	90	0	0	10	
15	M	50	01	LUL	0.5	N	AP	0	100	0	0	0	
			02	LUL	2.0		LP	55	45	0	0	0	
16	M	71	01	RUL	2	N	AP	0	60	20	0	20	
			02	RUL	0.8		AIS	100	0	0	0	0	
			03	RUL	0.5		AIS	100	0	0	0	0	
			04	RUL	0.8		AP	0	100	0	0	0	
17	M	63	01	RML	1.2	N	LP	70	30	0	0	0	
			02	RML	1		SP	0	20	0	0	80	
18	M	54	01	RML	1	Y	SP	0	30	30	0	40	
			02	RLL	0.4		LP	90	10	0	0	0	
19	F	65	01	RUL	1.2	Y	AP	0	60	40	0	0	
			02	RLL	1.1		AIS	100	0	0	0	0	
20	F	60	01	RLL	2.5	Y	AP	30	70	0	0	0	
			02	RUL	4		SP	0	40	0	0	60	
21	M	63	01	RLL	1.0	Y	SP	0	10	10	0	80	
			02	RML	0.5		AP	20	80	0	0	0	

Abbreviations: *A* acinar, *AIS* adenocarcinoma in situ, *AP* acinar predominant, *ASC* adenosquamous carcinoma, *F* female, *L* lepidic, *LLL* left lower lobe, *LM* Lymph node metastasis, *LP* lepidic predominant, *LUL* left upper lobe, *M* male, *M* micropapillary, *MA* mucinous adenocarcinoma, *N* none, *P* papillary, *PP* papillary predominant, *RLL* right lower lobe, *RML* right middle lobe, *RUL* right upper lobe, *S* solid, *SP* solid predominant, *Y* yes

### Histological assessment

Specimens were fixed using 10% neutral buffered formalin, paraffin embedded, and then stained with hematoxylin and eosin (HE). The histological subtypes of lung adenocarcinomas were evaluated using the 2015 WHO classification criteria [5]. Each histological component was recorded in 5% increments. The tumours were categorized by their main pattern: lepidic predominant adenocarcinoma (LP), acinar predominant adenocarcinoma (AP), papillary predominant adenocarcinoma (PP), micropapillary predominant adenocarcinoma (MP), and solid predominant adenocarcinoma (SP).

CHA and nonmucinous lepidic components were used as complementary approaches. SMLC can be considered SMPLC if the following four criteria are met: (1) differences in major histology subtype, (2) at least one tumours has beeen diagnosed as AIS, (3) a low grade lepidic component is present in all tumours, (4) similar major histology subtype but differences in other histology subtypes.

### Genomic DNA extraction and gene mutation analysis

DNA was extracted from sections of the FFPE. Target tumours lesions and control tissues were evaluated by pathologists. A DNeasy Blood and Tissue Kit was used to isolate genomic DNA (Qiagen, Hilden, Germany). Gene mutations (EGER, KRAS, BRAF, NRAS, ALK, ROS1, RET, HER2, and PIK3CA) were analysed using an amplification refractory mutation system (ARMS) with a gene mutation detection kit (Amoy Diagnostics Co, Xiamen, China).

### Statistical analysis

Statistical analyses and data visualization were performed using IBM SPSS Statistics version 22.0 (IBM SPSS, Inc., Chicago, IL, USA). The correlation between drive gene mutations and tumour lesion size was determined by Pearson’s correlation test.

## Results

### Clinical characteristics of SMLC patients

The clinical characteristics of the 21 selected patients are summarized in Table 1. There were 10 men and 11 women. The mean age was 60.5 years, and the ages ranged from 32 to 87 years. Among the 21 patients, 16 had two lesions, two had three lesions, and three had four lesions. Tumours occurred in both lobes in 10 patients. Lymph node metastases were present in four patients. Fifty tumours were examined in this study: nine occurred in the left lung, and 41 occurred in the right lung. There was one adenosquamous carcinoma and 49 adenocarcinomas, which included one mucinous adenocarcinoma. The tumour diameter ranged from 0.5 to 4 cm.

### Morphological and immunohistochemical assessment

**Fig. 1:**
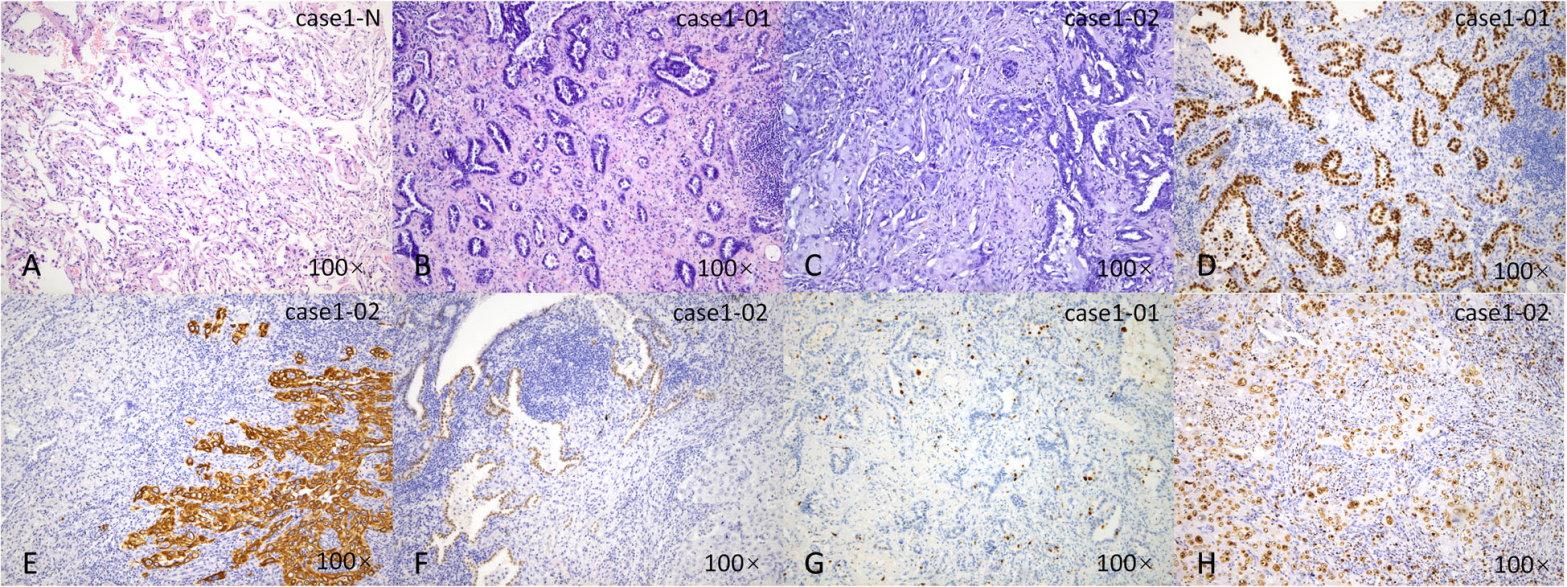
Classification of a case of SMPLC accordomg to the Martini and Melamed criteria. Normal lung tissue (**a**, HE), an adenocarcinoma in the left upper lobe (**b**, HE; **d**, TTF-1; **g**, ki-67) and an adenosquamous carcinoma in the right upper lobe (**c**, HE; **e**, CK5/6; **f**, TTF-1; **h**, ki-67) of case 1

**Fig. 2:**
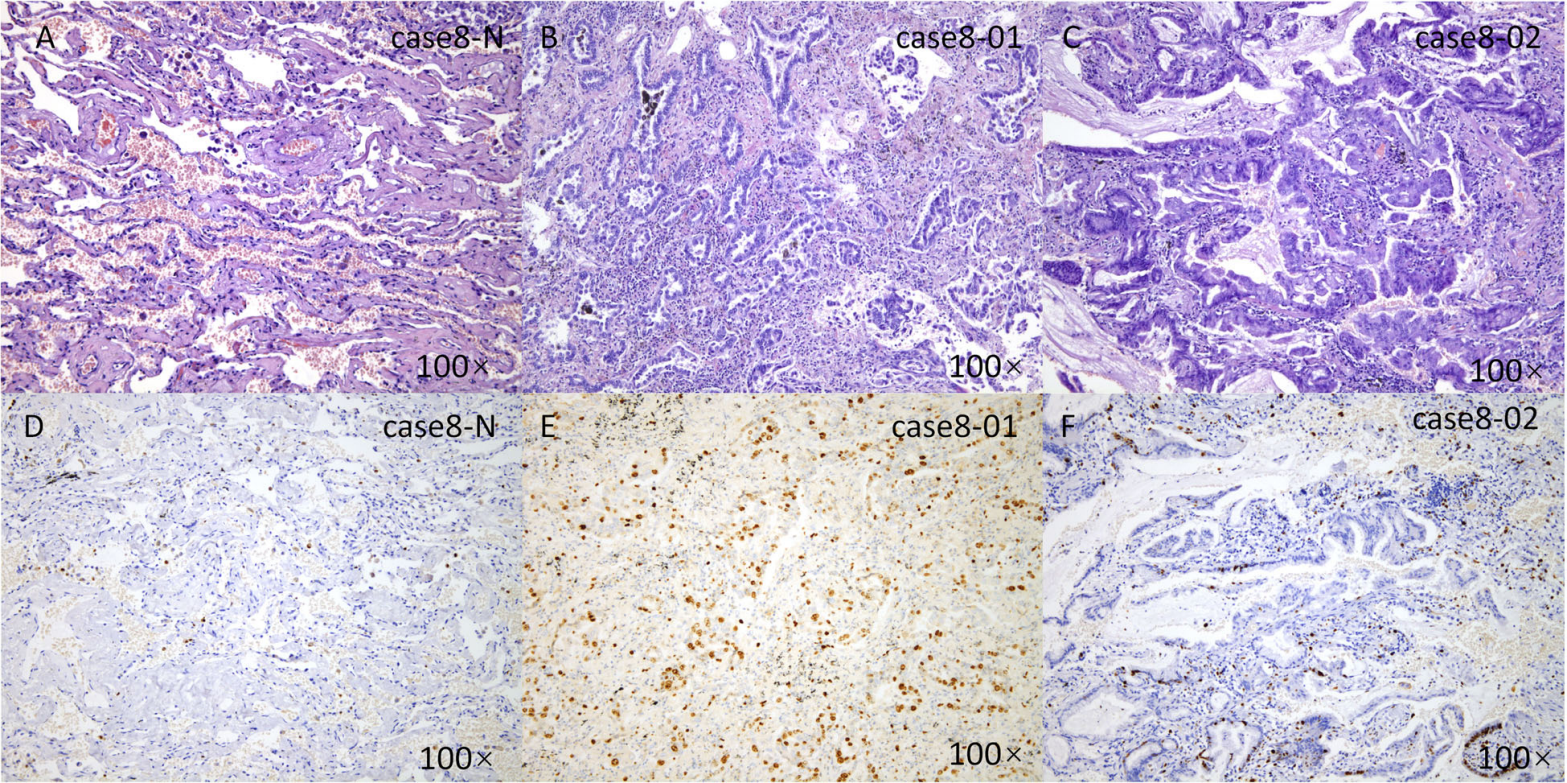
Classification of a case of SMPLC according to the Martini and Melamed criteria. Normal lung tissue (**a**, HE; **d**, ki-67), an adenocarcinoma in the right upper lobe (**b**, HE; **e**, ki-67) and a mucinous adenocarcinoma in the right lower lobe (**c**, HE; **f**, ki-67) of case 8

**Fig. 3:**
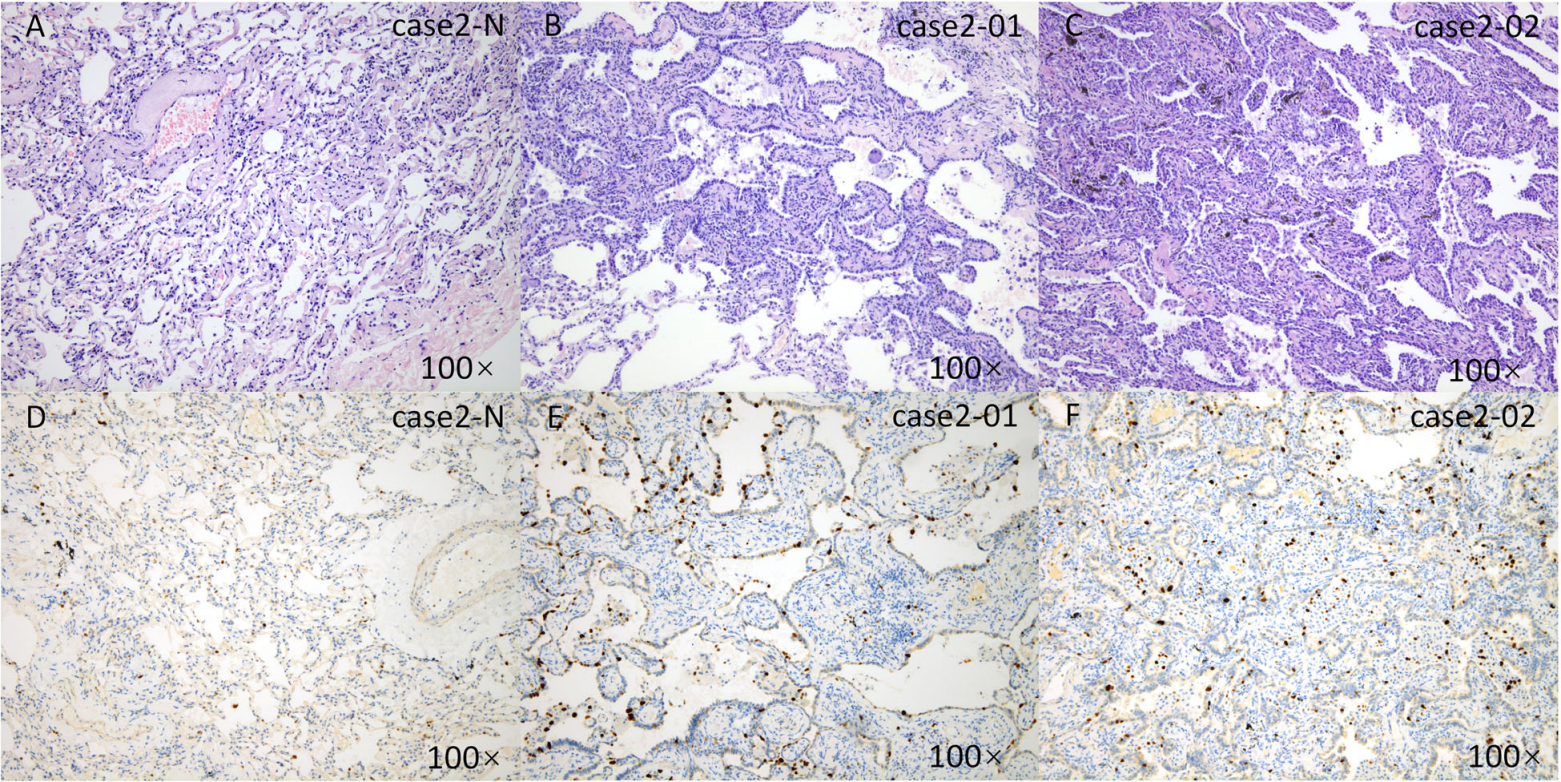
Classification of a case of SMPLC according to the Martini and Melamed criteria. Normal lung tissue (**a**, HE; **d**, ki-67) and two separate adenocarcinoma foci in situ in the left lower lobe (**b**, and **c**, HE; **e**, and **f**, ki-67) of case 2

According to the Martini-Melamed classification, 8 cases were considered SMPLC. In case 1, the histological types of the two lesions differed: adenocarcinoma and adenosquamous carcinoma (Fig. 1). In case 8, the histological types were adenocarcinoma and mucinous adenocarcinoma (Fig. 2). In case 2, both lesions were AIS (Fig. 3). In the remaining 5 cases, the lesions were located in both lung lobes, and no lymph node or distant metastasis was identified.

**Fig. 4:**
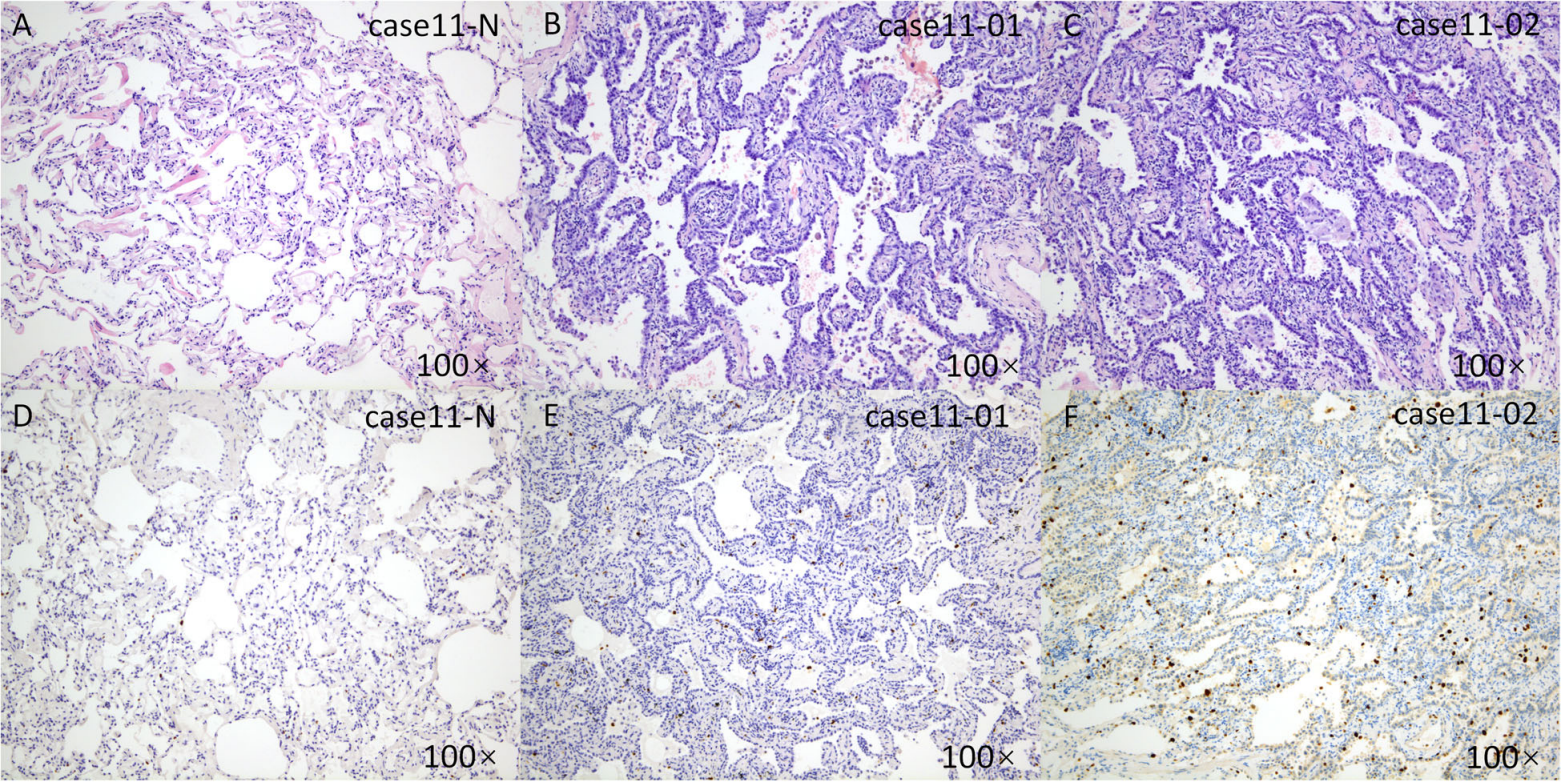
Classification if a case of SMPLC according to the refined standard. Normal lung tissue (**a**, HE; **d**, ki-67), an AIS (**b**, HE; **e**, ki-67) and a lepidic predominant adenocarcinoma lesion (**c**, HE; **f**, ki-67) in the right lower lobe of case 11

According to the refined standard, nine of the 13 cases that were originally classified as IM, were SMPLCs. Three of these cases (cases 9 to 11) contained two lesions, one of which was AIS. There were three and four lesions in cases 12 and 13, respectively; each lesion contained either a nonmucinous lepidic component or was just AIS. The histological subtypes of multiple lesions all differed in the remaining four cases (Fig. 4).

The remaining four cases were classified as IM due to lymph node metastasis. Overall, of the 21 tumour pair comparisons, 17 (81%) were independent primaries and four (19%) were related metastases.

Immunohistochemical testing showed that the adenocarcinoma components were positive for TTF-1, Napsin A, and CK7 and negative for CK5/6 and p40. In contrast, the Ki-67 index was only related to the histological subtypes of a lesion and was not related to the case or whether the lesion was primary.

### Mutational profiling

Fifty lung carcinomas from 21 patients were screened for mutations in EGFR, KRAS, BRAF, NRAS, ALK, ROS1, RET, HER2, and PIK3CA using ARMS. Thirteen cases with at least one sample had a point mutation or rearrangement. Of the 50 successfully tested carcinomas, EGFR mutations were identified in 16 cases (32%; two in exon 18, four in exon 19, two in exon 20, eight in exon 21), and KRAS mutations were identified in two (4%; two exon 2). No BRAF, NRAS, ALK, ROS1, RET, HER2 or PIK3CA mutations were identified. No mutation was identified in 32 of the 50 (64%) screened tumours.

Eight cases had a diagnosis of SMPLC according to the Martini-Melamed criteria (cases 1–8). Among these cases, three had wild-type oncogenes (case 1–3), and the lesions were the same. The remaining 5 cases had different gene mutations.

According to CHA, cases 9 to 17 can be interpreted as SMPLC. Cases 9 to 11 all contained two lesions, one of which was AIS. Both lesions of case 9 were wild-type. The other two cases had different molecular characteristics. In case 10, the AIS had a KRAS mutation, while no mutation was identified in the solid lesions. In case 11, EGFR mutations were identified in both lesions, but their specific sites differed.

In cases 12 and 13, the multiple foci were either in situ carcinoma or contained nonmucinous lepidic components. Case 12 had three lesions, which included two mutations in EGFR exon 21 (L858R), while the AIS lesion was wild-type EGFR. Case 13 had four lesions, of which three lesions had mutations at various sites within the EGFR gene, and one lesion was wild type, thereby suggesting different origins of the multilesion clones.

Not all lesions in cases 14–17 had lepidic components, but those without were of different histological subtypes. Only two lesions in case 14 had incongruent molecular characteristics. One of the two lesions was wildtype, and the other had mutations in EGFR exon 18 (G719X). In cases 15–17, as no driver gene mutations were detected, the clonal origin of the multiple lesions could not be determined.

Cases 18–21 were diagnosed as IM due to lymph node metastasis. In case 18, both lesions were wildtype. Both lesions of case 19 had an L858R mutation in EGFR exon 21. The right middle lesion of Case 20 had an EGFR exon 18 (G719X) mutation. The lower lesion was wildtype. Case 21 had a KRAS (G12DS) mutation in the right middle lesion, while the lower nodule was wildtype.

**Fig. 5:**
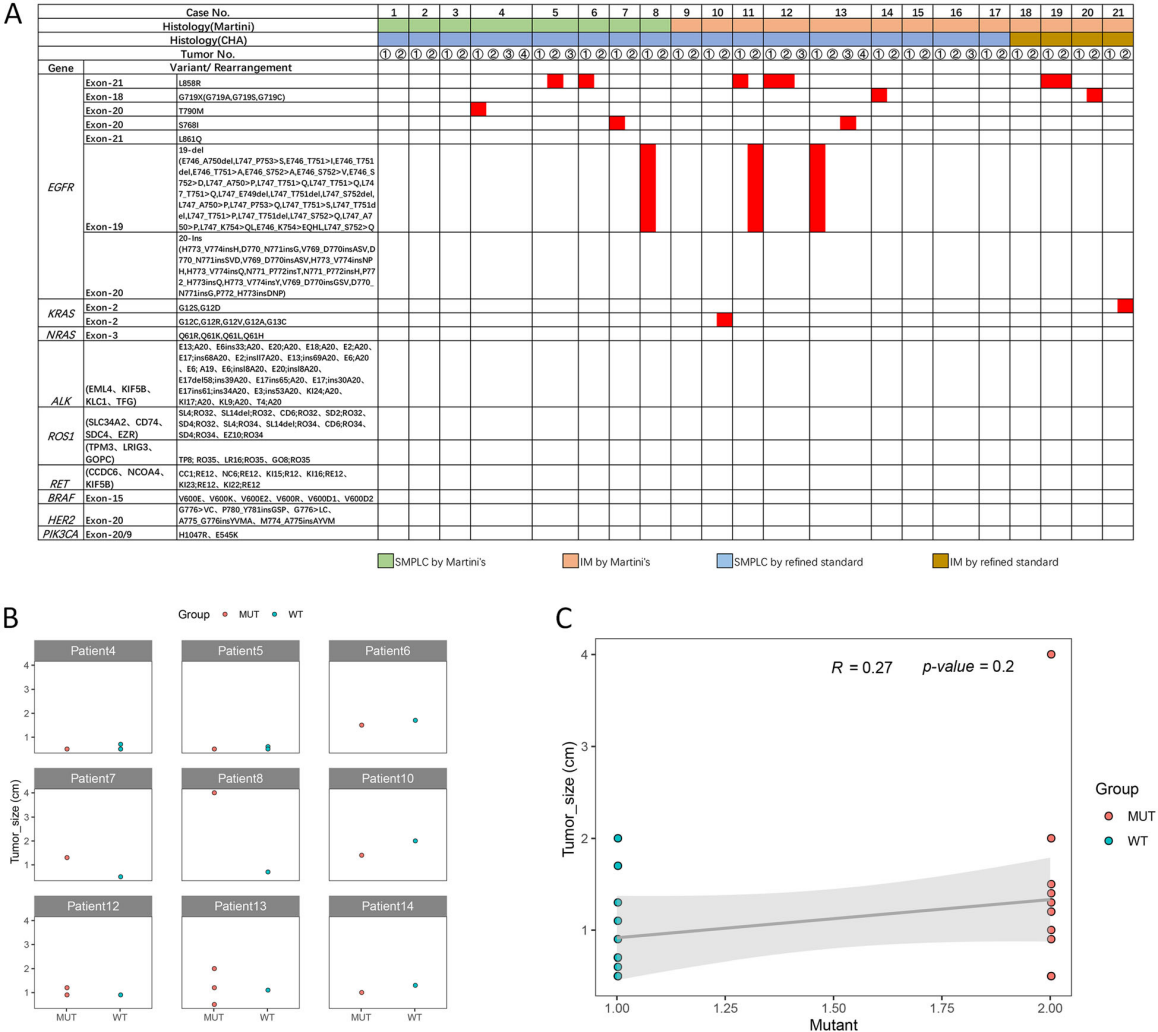
Mutation analyses of 50 tumours from 21 patients. **a** shows the genetic mutation status of each tumour. The red columns indicate the presence of mutations. Gene mutations were identified in 18 tumours (36%). **b** and **c** compare the tumour sizes between driver mutation-positive and driver mutation-negative lesions

In seven of the 17 SMPLC cases, there were no mutations in any lesion. Seven cases had different mutation statuses among lesions. In the remaining 3 cases, mutations in driver genes were identified in some lesions but not all (see Fig. 5a).

To explore the role of driver genes in SMLC, we compared the sizes of the lesions with positive and negative driver mutations in SMLC patients and found that the lesions with positive driver genes were not larger than those with negative driver mutations in the same patient, as shown in Fig. 5b. A statistical analysis (PCC, Pearson correlation coefficient) was conducted on the sizes of the lesions with positive and negative driver genes in the above patients, and no significant difference in lesion size between the two groups was identified (*P* = 0.27) (Fig. 5c).

## Discussion

The pathological assessment of multiple pulmonary nodules is crucial when distinguishing SMPLC from IM. Differentiation of biologically unrelated SMPLC from IM leads to accurate prognosis and helps guide treatment. The Martini-Melamed criteria were of limited use, since they considered only the major histologic tumour types: adenocarcinoma versus squamous cell carcinoma. An accurate histological evaluation should also include the relative proportion of each histologic subtype.

In our previous study, 164 patients with multifocal lung cancer were grouped, and their outcomes were analysed. We identified no significant difference in overall survival or disease-free survival between patients who were grouped according to the Martini-Melamed criteria. Survival was only significantly related significantly to tumour size, thereby suggesting that this classification system is of limited prognostic value in SMLC [8]. Nicholson et al. [9] had an international panel of lung pathologists conduct a detailed assessment of histological characteristics using the criteria of Martini and Melamed. These researchers identified the following set of features: histological subtypes (predominant and minor histologic patterns), size and pleomorphism of the nucleus, acinar structure formation, nucleoli size, and pathological mitosis rates. These researchers did not compare their results to molecular cloning or immune indicators. One study indicated that the whole spectrum of adenocarcinoma with lepidic components (AIS or lepidic predominant adenocarcinoma) has a favourable prognosis [10]. Thus, except for AIS, minimally invasive adenocarcinoma (MIA) and lepidic growth-dominated invasive adenocarcinoma should be regarded as unrelated primary tumours. According to Sun et al. [11], nonmucinous lepidic components with mild nuclear atypia (NLCMA) suggest primary lesions. These researchers sought to evaluate this conjecture by retrospectively analysing 116 lesions from 54 patients using a combination of the CHA and NLCMA criteria, with statistical results indicating a significant difference in disease-free survival after grouping.

Pathologists view similar histological patterns as only relative arguments in favour of homologous tumour sources. In evaluating multifocal lung cancer, pathologists should seek an appropriate balance between molecular detection and histological features of the tumour. Recently, several studies have been reported in which molecular biology techniques are used to analyse SMLC. These techniques include comparative genomic hybridization (CGH), DNA microsatellite analysis, and next-generation sequencing (NGS) [12–14]. Tumors with similar molecular characteristics are posited to be IM with a monoclonal origin. Discordant tumours are posited to be independent primary tumours. The discrepancies between histopathological and molecular SMPLC classifications in various cohorts range between 18 and 30% [15].

Many studies have defined tumours with specified driver mutations as monoclonal [16]. Driver mutations can be used in clinical applications to determine tumour lineages as successfully as histological and clinical reviews, but may lead to misclassification in challenging cases [17–19]. We must be extremely careful in our interpretation.

First, the same genetic mutation should occur in morphologically different lesions [20]. Another problem is the heterogeneity of mutations in primary tumours and metastatic lesions, especially EGFR and KRAS, with the misclassification rate ranging from 0 to 45% [21, 22]. Moreover, driver mutations also occur in normal-appearing lungs in paracancerous tissues [23]. Second, data show that known driver mutations cannot be detected in approximately 50% of lung cancers, and thus cannot provide useful predictions [24–26]. Third, multiple driver mutations in a single NSCLC tumour rarely occur, and it is difficult to extrapolate from a single mutation in a single case.

It is clear that similarity of gene mutation lineages among lesions, including the same mutation or wild-type, does not necessarily indicate a clonal relationship. Likewise, different gene mutation lineages among multiple lesions do not indicate different primary origins.

Even the integration of clinical assessment, histology, and the presence of driver mutations is not sufficient for accurate prediction if multifocal tumours have the same origin [27]. More objective methods and additional data are required to address this problem.

We collected 21 cases of SMLC and analysed their histological subtypes. The cohort included explicit SMPLC cases that conform to the Martini-Melamed criteria, cases with a nonmucinous adherence component as defined by the improved criteria, and explicit cases of IM. Their molecular properties were analysed further; eight cases had no driver gene mutation, and the clonal origin could not be determined. Of the eight cases that were classified as SMPLC using the Martini-Melamed criteria, driver mutations were not identified in three. Of the other five cases, four had two lesions, and a gene mutation was identified in one focus, thereby suggesting different clonal origins. In the remaining case, there were four lesions: an EGFR T790M mutation was identified in one, and no driver mutations were detected in the other three. Thus, the use of gene mutations to define clonal origin is of limited value.

In our cohort, nine cases were identified as SMPLC using our recently revised organizational standard rather than the Martini-Melamed criteria. The lesions of these cases all occurred in the same lung lobe, and not all the lesions were AIS. However, a careful analysis of the histological subtypes showed that all lesions contained nonmucinous lepidic lung cancer components. An analysis of gene mutations indicated that four cases had no driver mutations, and it was of limited value for determining if they were primary or not using gene mutations.

The remaining four cases were consistent with the known genetic characteristics of SMPLC: the mutation sites were different or there was a mutation from wild-type in only one lesion. However, the same mutation was identified in two lesions of case 12 (L858R in EGFR exon 21). According to the histomorphology, the histological subtypes of the two lesions were similar, and they were mainly composed of lepidic and acinar types, without complex components such as papilla, micropapilla or solid patterns, thereby rendering challenging the determination of the clonal origins of these lesions.

However, interfocal molecular characteristics may also differ in cases that are identified histologically as IM. The two lesions of case 18 were wild-type, and the gene mutations of both lesions of case 19 were L858R in exon 21 of EGFR. The mutation profiles of these cases suggested that the multiple lesions were derived from the same clone. However, in the other two IM cases, no mutations were identified in the acinar-type lesions of case 20, while EGFR gene mutations were detected in the other solid-type lesion. Only one lesion of case 21 had a KRAS mutation. Consistent with cases that were reported in the literature, this is indicative of clear inconsistencies between molecular and histological characteristics when studying multifocal lung cancer.

In addition, driver gene mutations are known to be associated with faster tumour cell growth. Interestingly, however, our study found that in SMLC, there was no unique growth advantage in lesions with positive driver genes, and there was no significant difference in lesion size between lesions with positive and negative driver genes. This may be a unique feature of SMLC, but it may also be because the wild-type group carries other driver gene mutations that are not within our detection range; thus, we will expand the detection range for further verification in the future.

It is often challenging to definitively determine whether SMLPs are independent primary or intrapulmonary metastases. The classification of difficult cases requires a detailed histological analysis and molecular characterization and often benefits from multidisciplinary discussions among oncologists, pathologists, and surgeons. The increasing popularity of NGS and more comprehensive whole-exome sequencing should ultimately increase the accuracy of interpretation.

## Conclusions

In the 21 patients with SMLC in our study cohort, whether the mutation sites were the same did not directly indicate the clone origins of foci of SMLC. The molecular and histological characteristics of SMLC are not completely consistent. Therefore, our results suggest that the use of the presence of nonmucinous lepidic components as a sign of a primary tumour usefully complements the traditional histological classification of multifocal lung cancer. Moreover, it is necessary to identify and even sequence driver mutations in each lesion. This can play a key role in staging and grading multifocal lung cancer patients, thereby directly affecting the targeted treatment regimens. For the clinical stage assessment of patients with multifocal lung cancer and the formulation of suitable treatment plans, case analysis and precise personalized treatment are required.

## Data Availability

The datasets used and /or analyzed during the current study are available from the corresponding on reasonable request.

## Abbreviations

AP: acinar predominant adenocarcinoma
ARMS: amplification refractory mutation system
CGH: comparative genomic hybridization
CHA: comprehensive histological assessment
CT: computed tomography
HE: hematoxylin and eosin
IM: intrapulmonary metastasis
LP: lepidic predominant adenocarcinoma
MIA: minimally invasive adenocarcinoma
MP: micropapillary predominant adenocarcinoma
NGS: next-generation sequencing
NLCMA: nonmucinous lepidic components with mild nuclear atypia
PP: papillary predominant adenocarcinoma
SMLC: synchronous multifocal lung cancer
SMPLC: synchronous multifocal primary lung cancer
SP: solid predominant adenocarcinoma
